# Deprescribing in Older Poly-Treated Patients Affected with Dementia

**DOI:** 10.3390/geriatrics9020028

**Published:** 2024-02-26

**Authors:** Pietro Gareri, Luca Gallelli, Ilaria Gareri, Vincenzo Rania, Caterina Palleria, Giovambattista De Sarro

**Affiliations:** 1 Department of Frailty, Center for Cognitive Disorders and Dementia (CDCD) Catanzaro Lido—ASP Catanzaro, Magna Graecia University, 88100 Catanzaro, Italy; 2 Unit of Clinical Pharmacology and Pharmacovigilance, “Renato Dulbecco” University Hospital, 88100 Catanzaro, Italy; gallelli@unicz.it (L.G.); raniavincenzo1@gmail.com (V.R.); desarro@unicz.it (G.D.S.); 3 Department of Health Science, Magna Graecia University, 88100 Catanzaro, Italy; ilariagareri1@gmail.com (I.G.); palleria@unicz.it (C.P.); 4 Research Center FAS@UMG, Department of Health Science, Magna Graecia University, 88100 Catanzaro, Italy

**Keywords:** deprescribing, older patients, dementia, university, territory, poly-treatment

## Abstract

Polypharmacy is an important issue in older patients affected by dementia because they are very vulnerable to the side effects of drugs’. Between October 2021 and September 2022, we randomly assessed 205 old-aged outpatients. The study was carried out in a Center for Dementia in collaboration with a university center. The primary outcomes were: (1) deprescribing inappropriate drugs through the Beers and STOPP&START criteria; (2) assessing duplicate drugs and the risk of iatrogenic damage due to drug–drug and drug–disease interactions. Overall, 69 men and 136 women (mean age 82.7 ± 7.4 years) were assessed. Of these, 91 patients were home care patients and 114 were outpatient. The average number of the drugs used in the sample was 9.4 drugs per patient; after the first visit and the consequent deprescribing process, the average dropped to 8.7 drugs per patient (*p* = 0.04). Overall, 74 potentially inappropriate drugs were used (36.1%). Of these, long half-life benzodiazepines (8.8%), non-steroidal anti-inflammatory drugs (3.4%), tricyclic antidepressants (3.4%), first-generation antihistamines (1.4%), anticholinergics (11.7%), antiplatelet drugs (i.e., ticlopidine) (1.4%), prokinetics in chronic use (1.4%), digoxin (>0.125 mg/day) (1.4%), antiarrhythmics (i.e., amiodarone) (0.97%), and α-blockers (1.9%) were included. The so-called “duplicate” drugs were overall 26 (12.7%). In total, ten potentially dangerous prescriptions were found for possible interactions (4.8%). We underline the importance of checking all the drugs taken periodically and discontinuing drugs with the lowest benefit-to-harm ratio and the lowest probability of adverse reactions due to withdrawal. Computer tools and adequately trained teams (doctors, nurses, and pharmacists) could identify, treat, and prevent possible drug interactions.

## 1. Introduction

The progressive increase in life expectancy recorded in the last few years has been associated with an overall improvement in the quality of life in old age but has also led to an increase in the number of people with disabilities, chronic diseases, and prescription drugs [1].

The number of drugs taken daily increases with age and the number of prescribers [2]. Therefore, the poly-treated patient is usually an older patient who uses five or more drugs with an increased risk of adverse drug reactions (ADR). According to a survey conducted four years ago in Canada, one out of two Canadians over the age of 65 take at least five prescription drugs, and one out of four take more than 10 drugs, of which 20% are between 65 and 74, 32% between 75 and 84, and 39% are 85 years of age or more [3].

In a multidisciplinary team composed of a geriatrician, a clinical pharmacologist, and a family doctor, it is important in clinical management to periodically check all of the therapies taken by a patient and to avoid possible iatrogenic damage.

However, notwithstanding the large efforts to develop criteria for assessing medication appropriateness in older people in recent years, few studies focused on people with dementia [4]. People affected by dementia and comorbidities are a huge problem both in old outpatient and in hospitalized or residential patient drug prescriptions, because old age and cognitive impairment make them extremely vulnerable to various drugs side effects [5]. In a recent systematic review and meta-analysis, 62 eligible studies were included, of which 53 studies reported the prevalence of potentially inappropriate medications (PIM), and 28 studies reported the prevalence of polypharmacy. In particular, the pooled estimate of PIM and polypharmacy was 43% (95% CI 38–48) and 62% (95% CI 52–71), respectively [5].

The most widely used validated tools to prevent the use of potentially inappropriate drugs (PIMS—Potentially Inappropriate Medications) in older patients are the Beers criteria; they were introduced in 1991 and repeatedly revised in 2012 and 2015. The last revision, made by the American Geriatric Society, was in 2019 [6].

**Figure 1:**
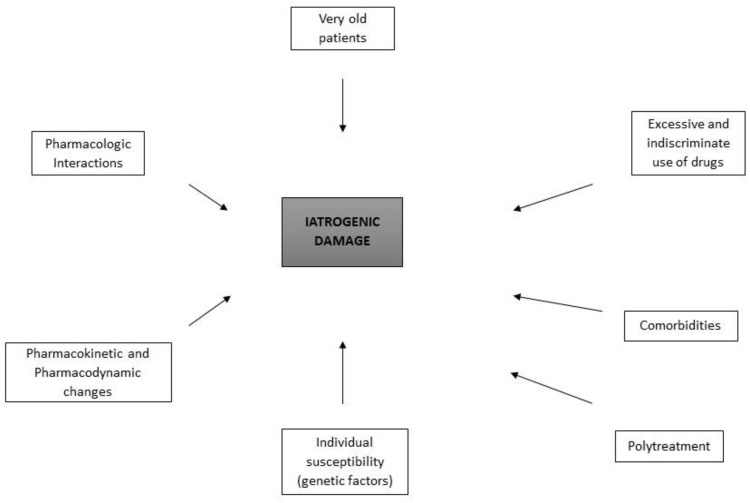
Summarizes the possible factors related to iatrogenic damage.

The STOPP (Screening Tool of Older Persons’ potentially inappropriate Prescriptions) and START (Screening Tool to Alert Prescribers to Right Treatment) criteria were developed in order to have tools available to avoid potentially harmful prescriptions [7,8]. Figure 1 reports the possible factors leading to harmful events due to drugs.

Deprescribing is “a systematic process of identifying and discontinuing drugs or pharmacological regimens in circumstances where obvious or potential adverse effects outweigh their current and/or potential benefits, taking into account treatment objectives, treatment safety (Table 1), level of functioning, life expectancy, values, and preferences of the individual patient” [9]. Indeed, drugs with a narrow therapeutic index could be dangerous, because the therapeutic dosage is very close to the toxic dose.

Potentially inappropriate medications (Table S1), inappropriate drugs according to class and/or dosage (Table S2), and potential interactions according to the number of drugs (Table S3) are reported in Supplementary Data.

The aim of the present study was to evaluate geriatric, poly-treated outpatients assessed at a Center for Cognitive Disorders and Dementia, in collaboration with the Deprescribing Clinic at the Unit of Clinical Pharmacology and Pharmacovigilance of a University–Hospital of Catanzaro.

## 2. Methods

This study was conducted at the Center for Cognitive Disorders and Dementia (CDCD) of Catanzaro Lido and the Unit of Clinical Pharmacology and Pharmacovigilance, “Renato Dulbecco” University–Hospital of Catanzaro, on outpatients or home patient’s follow up.

A multidimensional geriatric assessment was carried out for each patient, with the application of some tests. Renal function was evaluated through the glomerular filtration rate (GFR), applying the CKD-EPI equation [10].

The study evaluated two populations of dementia patients, outpatients, and home patients, for the time between 1 October 2021, and 30 September 2022. This study was part of the PharE (Pharmacovigilance in the Elderly) project, an ongoing study in Italy, involving elderly patients in ambulatory, home, and residential care settings; a part of the study is being dedicated to people affected with dementia. Previously, we studied 972 residential care patients in a three-year time frame [11], whereas the present study was carried out on home and ambulatory care patients in a one-year time frame.

Overall, 205 patients, 114 outpatients, and 91 home patients, both female and male, were randomly included and analyzed. An Excel file was created for recording all the patients, ages, genders, settings where the visit was performed (ambulatory, home), and the kind of drugs used. The geriatricians were responsible for the treatment, in other words, they signed the prescriptions. The patients visited could have been visited by other specialists and/or by the general practitioner. The medication review was made by geriatricians and pharmacologists of the University Center for Deprescribing. The changes were sent in a letter to the general practitioner following the visit and the medication review. 

The study included the collection of data for each patient as follows:

- Personal data: age, sex, and kind of patient—if outpatient or at home.
- Clinical-pathological data: identification of the main diagnosis, and the possible co-morbidities.
- Cumulative Illness Rating Scale (CIRS): this is a valuable tool to indicate the health status of older, frail subjects. We can obtain two indices, severity and comorbidity, which are effective in predicting outcomes of hospitalization, disability, and mortality. The severity index results from the average of the scores obtained by considering 13 categories concerning the functionality of all organs and apparatus excluding psychiatric–behavioral pathologies. The score ranges between 1 (absence of pathology) up to a maximum of 6 (very serious pathological condition). The comorbidity index represents the number of categories in which a score greater than or equal to 3 is obtained, also excluding in this case psychiatric–behavioral pathologies. The doctor, based on the clinical history, the objective examination, and the present symptomatology define the level of severity of the pathological conditions of the patient [12].
- Functional assessment scales: ADL (Activity of Daily Living) and IADL (Instrumental Activity of Daily Living) both specify the level of activity that the older person is able to perform.
- Cognitive Assessment scales: MMSE (Mini-Mental State Examination), a test for the assessment of cognitive efficiency and the presence of cognitive impairment. It is based on an assessment scale from 0 (maximum cognitive deficit) to 30 (no cognitive deficit). About the score obtained, a classification is performed:
  - 26–30—normal condition;
  - 25 borderline;
  - 18–24 mild to moderate impairment;
  - 13–17 medium-severe cognitive impairment;
  - <13 severe deterioration.

In addition, the total score is adjusted according to the age and educational attainment of the individual examined [13].

- Pharmacological history: The pharmacological therapy of each patient was fully evaluated and for each drug indicated, the category, the active substance, the prescribed dose, and the frequency of administration was recorded. Over-the-counter products such as supplements have also been included because they can be responsible for drug interactions, including serious interactions. Finally, the total number of drugs taken by the patient was analyzed. In the case of suspected ADR, Naranjo’s algorithm was applied [1,14]. The study of possible drug–drug interactions was carried out using Micromedex 2.0 [15].
- Evaluation of potentially inappropriate drugs: using the Beers and STOPP&START criteria, the possible intake of potentially inappropriate drugs was evaluated. The STOPP&START criteria were used to facilitate the deprescribing process or, in any case, to address patients’ treatment correctly. Inappropriate or potentially inappropriate drugs, duplicate drugs, resulting from possible prescriptive errors or lack of control of therapy, and drugs inducing potentially dangerous interactions were assessed.

The primary outcomes were:

- Encouraging drug deprescribing by limiting the use of inappropriate drugs through the use of Beers and STOPP&START criteria [1,6,7,8,16];
- Evaluating the possible presence of duplicate drugs, or in the context of polypharmacy, the risk of iatrogenic damage for drug–drug and drug–disease interactions.

The secondary outcomes were:

- Personalizing treatment to reduce ADR and consequently the indirect costs for health;
- Developing possible suggestions to target the pharmacological management of the poly-treated patient as correctly as possible.

All subjects gave their informed consent for inclusion before they participated in the study. The study was conducted in accordance with the Declaration of Helsinki. The study protocol was approved by the Ethics Committee (Italy, Calabria Region, registered protocol number 29—11 February 2016—Clinical Study “Assessment of the use on inappropriate drugs, adverse reactions and the possible pharmacological interactions in elderly poly-treated people—The PharE Study).

### Statistical Analysis

Data were expressed as mean ± standard deviation (DS). The ANOVA test was used to assess the differences between two groups (for example, users of an inappropriate drug versus non-users). The differences were classified as significant for values of *p* < 0.05.

## 3. Results

**Figure 2:**
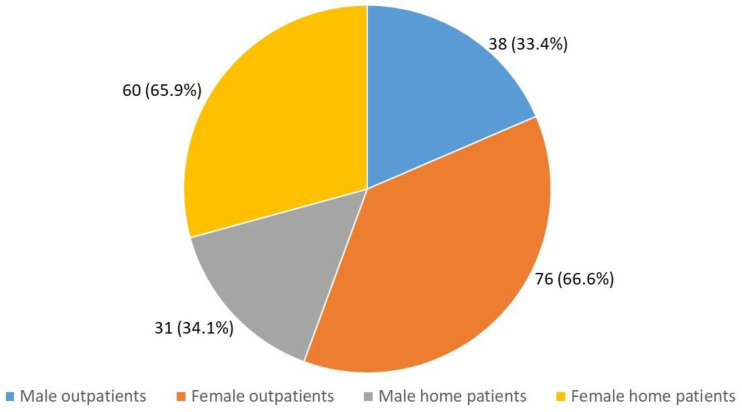
Distribution of the sample according to gender and care setting.

We evaluated 205 patients, 69 men (33.6%) and 136 women (66.4%), with a mean age of 82.7 ± 7.4 years. In particular, 91 patients of these, 31 men (34.1%) and 60 women (65.9%) were home patients, and 114 were outpatients, 38 men (33.4%) and 76 women (66.6%) [Figure 2]. 

The mean age was significantly higher in home patients (*p* < 0.001) [Table 2].

Table 2 shows the biographical, functional, cognitive characteristics, diagnoses, and drugs taken in the test sample.

The average of the drugs used in the sample was 9.4 drugs/patient (1936 prescriptions overall); after the visits, the deprescribed drugs were 135 overall, thus the average dropped after the first visit to 8.7 drugs/patient (*p* = 0.04).

The average of the drugs used was slightly higher in outpatients compared to home patients, although the difference was not statistically significant (*p* = 0.113; Table 2).

Home patients were more impaired in both cognitive, functional activities, and comorbidities. 

Since the study was carried out in a center for cognitive disorders, Alzheimer’s dementia was found in over one-fifth of outpatients, while mixed dementia (Alzheimer’s type dementia associated with cerebrovascular disease) was the most representative in home patients [Table 2]. 

The most frequent comorbidities were heart diseases (ischemic heart disease, hypertension, atrial fibrillation, heart failure) in both outpatients and home patients [Table 2].

**Figure 3:**
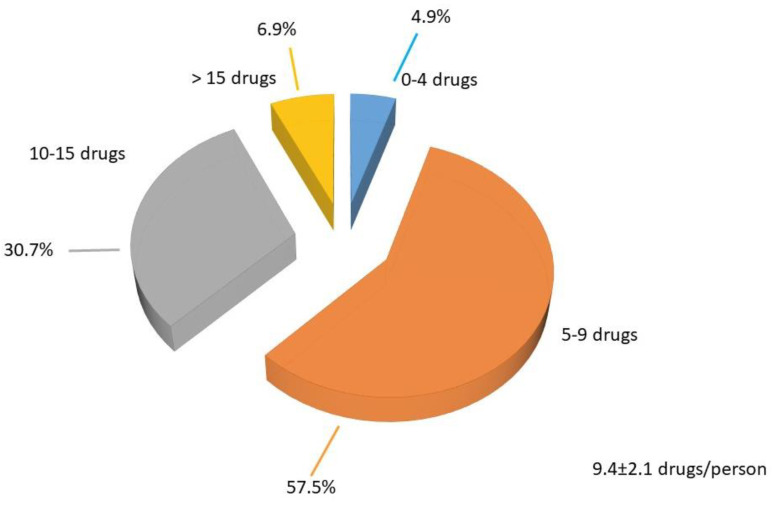
Drugs prescribed in the sample.

Figure 3 shows the drugs prescribed in the sample (0–4 drugs, 5–9 drugs, 10–15 drugs and >15 drugs). 

**Figure 4:**
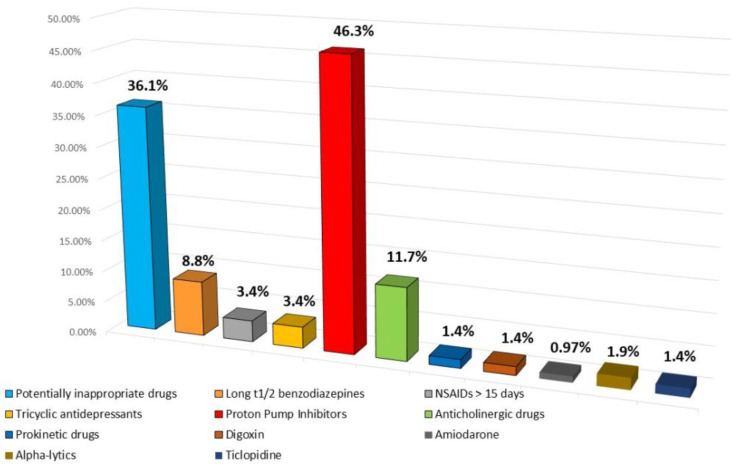
Drugs taken in the sample patients.

Figure 4 reports the kind of drugs taken in the sample patients.

### 3.1. Potentially Inappropriate Drugs

Potentially inappropriate drugs were 74 overall (36.1%). Of these, the long half-life plasma benzodiazepines were 18 (diazepam, N-desmethyl-diazepam) (8.8%), non-steroidal anti-inflammatory drugs in chronic use (>15 days, diclofenac sodium, ibuprofen, piroxicam, aceclofenac) were 7, (3.4%), tricyclic antidepressants (clomipramine, amitriptyline) were 7, (3.4%), first-generation antihistamines (hydroxyzine, oxatomide) were 3, (1.4%), anticholinergics (including both anticholinergic drugs such as benzodiazepines, tricyclic antidepressants, biperiden, fesoterodine) were 24, (11.7%), ticlopidine was 3 (1.4%), prokinetic drugs such as levosulpyride in chronic use over 20 days were 3 (1.4%), digoxin at dosage > 0.125 mg 3 (1.4%), amiodarone was 2 (0.97), and α-blockers (doxazosin, terazosin) were 4 (1.9%). 

### 3.2. Duplicate Drugs

The so-called duplicate drugs accounted for 26 overall (12.7%): two antidepressants were found in six patients (2.9%), two benzodiazepines were found in nine patients (4.4%), and three benzodiazepines simultaneously in one patient (0.5%). Overall, two antipsychotics together were found in six patients (2.9%), while in one patient, even the antipsychotics administered were three (haloperidol, promazine, quetiapine). In total, two α-blockers were found among the prescriptions in four patients (1.9%).

### 3.3. Drugs Potentially Dangerous for Interaction

In total, ten potentially dangerous prescriptions were found for interaction (4.8%): digoxin in patients with renal failure at a dosage > 0.125 mg (2 cases, 0.9%); three cases with risk of “triple whammy” (NSAIDs + ACE inhibitor/angiotensin-2 antagonists + furosemide) (1.4%), one case with risk of serotonin syndrome (SSRI + tricyclic + selegilin), three cases of pharmacodynamic antagonism (donepezil-biperiden, antidiarrheal/laxative, risperidone/L-DOPA), and one case of pharmacodynamic synergy (two sedative-hypnotic administered improperly together).

The total prescriptions for proton pump inhibitors were 95 (46.3%). In ten patients, proton pump inhibitors were interrupted as they had been taken for a long time without any need (patients were no longer being treated with potentially harmful gastrointestinal drugs and/or had no history of ulcerative pathology of the gastro-duodenal tract). 

In ten patients, over-the-counter drugs (mostly supplements of the osteoarticular system, polyvitamins) that were not considered necessary in the overall management of the patient’s therapy were deprescribed.

**Figure 5:**
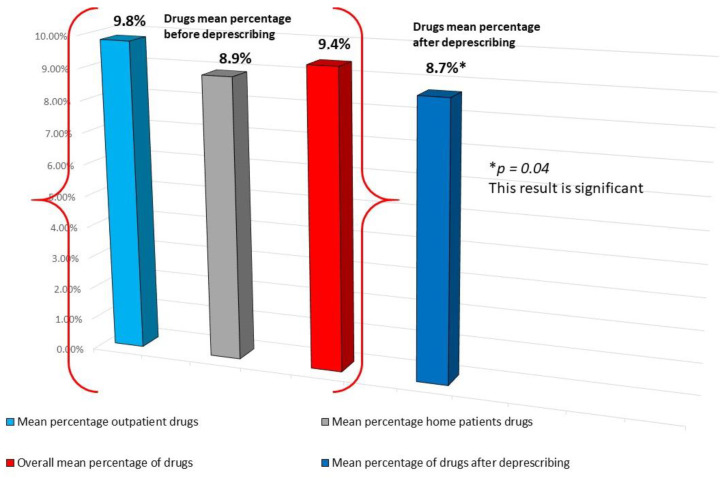
Drugs taken by patients of the sample before and after deprescribing.

Figure 5 shows the mean percentages of the drugs in outpatients and home patients and their percentage after deprescribing.

Figure 5 reports the drugs taken by patients of the sample before and after deprescribing.

## 4. Discussion

Safe prescription in older people is not easy due to comorbidities, and the increase in the number of prescribers favors overprescriptions and iatrogenic damage [1].

The guidance of a geriatrician as well as a clinical pharmacologist, in collaboration with a primary physician, is fundamental for complex older patients, and at the same time stimulating, as it is important to know the consequences of age-dependent changes on the kinetics and dynamics of drugs, drug metabolism, and the risk of harmful interactions (for example, drug–drug, drug–disease, herbal drugs, and over-the-counter products) [16,17]. Even OTC drugs are not at all easy to manage alone.

In our paper, the home population was significantly older, and it is also obvious to take into account that cognitive, functional, and behavioral disorders worsen from outpatients to home patients [Table 2], and even more among home and residential patients [11]. In total, 82% of the patients in the sample were taking more than five drugs, and even 14 patients (6.9%) took more than 15 drugs, including medicines, OTC, and herbal products. We found no significant differences in drug use among outpatients and the home population, and this could be related to what was said above, namely that walking patients make more visits with more specialists, who sometimes prescribe medications without considering the medications already taken by the patient as basic therapy. We did not find any gender differences in the use of drugs in our sample.

In addition, an 81-year-old outpatient came to our attention with 28 drugs prescribed by different specialists; two other patients, one 71-year-old and one 82-year-old, took 25 drugs at the first consultation. An 84-year-old patient took 19 drugs.

As mentioned above, 135 drugs were deprescribed, including inappropriate drugs, according to the Beers and STOPP&START criteria, duplicate drugs, and potentially dangerous drugs with a high risk of pharmacokinetic or pharmacodynamic interaction. More than one-third of the drugs were found to be potentially inappropriate, and among them, anticholinergic drugs ranked first.

Indeed, the central nervous system (CNS) is very sensitive to the anticholinergic side effects of drugs due to the substantial decrease in neurons or cholinergic receptors in the brains of older people.

The adverse effects of anticholinergics are mainly due to reduced kidney and liver function and increased blood–brain barrier permeability in older subjects. As a result, old patients may present with drowsiness or sedation, blurred vision, balance disorders, constipation, urinary retention (especially men suffering from benign prostatic hypertrophy), confusion or delirium, hallucinations, increased heart rate, xerostomia, reduced sweating, fever, and the risk of falls and consequent fractures [18,19].

Anticholinergic drugs can dramatically worsen the cognitive deterioration of dementia patients. In addition, a recent study showed that the high use of anticholinergic drugs is associated with an increased risk of dementia [20].

The excessive use of anticholinergic drugs (3 years or more) in all subclasses was linked to an increased risk of 54% for the development of dementia compared to taking the same dose of medication for three months or less [20]. Another interesting fact is that the results suggested that the risk of dementia due to anticholinergics remains even after discontinuation of the drug [21]. Tricyclic antidepressants and antihistamines also have anticholinergic properties.

Therefore, anticholinergic drugs should be avoided in older patients; among the drugs with high anticholinergic properties are biperiden, used in Parkinson’s disease (improperly in aged patients), first-generation antihistamines (oxatomide, hydroxyzine), and tricyclic antidepressants (amitriptyline, clomipramine).

Tricyclic antidepressants should be avoided in older patients, due to their pro-arrhythmic properties (a quinidine-like effect) and increase in QT interval [22].

As a result of the application of the START&STOPP criteria, we conveniently stopped anticholinergic, antihistamine, and tricyclic drugs in our sample.

Antihistamine drugs that had been prescribed for skin itching were replaced with new antihistamine drugs (desloratadine and bilastine). Whenever tricyclics had been prescribed to treat mood disorders, they were replaced with new antidepressant drugs, such as selective serotonin reuptake inhibitors (SSRIs) or selective norepinephrine reuptake inhibitors (NSARIs). If amitriptyline had been prescribed to improve sleep or treat chronic pain, it was replaced with a different sleep inducer or analgesic, respectively.

In our study, benzodiazepines had been prescribed to 69 patients (33.6%), and those with long plasma half-life had been prescribed to 18 patients (8.8%). However, long half-life benzodiazepines should be avoided in old people, especially in patients with cognitive impairment. In this study, diazepam, desmethyldiazepam, clonazepam, and flurazepam were stopped. Indeed, in a previous study, side effects and adverse events were more frequent in patients taking long half-life benzodiazepines than those not taking them (*p* = 0.0000); we reported paradoxical reactions, excessive sedation, fractures, and cognitive impairment [17]. Furthermore, several studies have widely demonstrated the increased risk of cognitive impairment and Alzheimer’s disease [22]. Therefore, it is advisable to limit or rather avoid the use of benzodiazepines; their use should be limited to a short time (it is often suggested “to start with the end in mind”), usually for anxiety control, together with antidepressants or in the treatment of insomnia.

Following the criteria of START&STOPP, we stopped long half-life benzodiazepines, replacing them with short half-life benzodiazepines, such as lorazepam or alprazolam, and then we also tried to stop their use. Lorazepam and alprazolam do not have a first-pass metabolism, which is reduced in older people, and are subjected only to glucuronide conjugation metabolism (phase II reaction), which does not undergo any substantial changes with age.

The benzodiazepines’ deprescribing is important in the geriatric population, although they have been a milestone in the treatment of anxiety disorders, since they replaced the dangerous barbiturates. The deprescription must be cautious, and a gradual suspension, especially in patients who have been taking this class of drugs for years, is recommended [1].

As for the inappropriate use of non-steroidal anti-inflammatory drugs (NSAIDs), their use should be extremely cautious. In any case, prolonged use exceeding 15 days must be avoided. NSAIDs are extremely harmful when associated with diuretics, ACE inhibitors, or blockers of the angiotensin receptor 2. The consequence of this dangerous combination of drugs is called the “triple whammy” and leads to acute renal failure and a potential risk of death [13,23,24].

The use of α-blockers was found in only 1.4% of patients; however, these drugs are harmful in older patients, especially for the orthostatic hypotension that they can cause.

Another important problem is duplicate prescriptions (we found doxazosin and terazosin together in four male patients) (1.9%). This may result from multiple prescribers because different specialists are used to prescribe the drug for the individual disease, where older patients have an incredible spectrum of comorbidities and multiple prescriptions.

In addition, the risk of pharmacodynamic enhancement occurs when one of these drugs is associated with drugs with α-lytic properties (for example, antipsychotics or older-generation antidepressants). In these cases, the risk of falls and fractures is very high and is a real problem for old patients [1,15].

We immediately eliminated the double prescription and replaced doxazosin with other antihypertensive agents, terazosin with more tolerable drugs, and especially with more selective α-blockers, such as tamsulosin and silodosin. However, we should take into account that, especially in patients with hypotension or normotension and under conditions of poor hydration, α-blocker drugs can still result in orthostatic hypotension in older patients.

As for the use of prokinetic drugs, these were prescribed inappropriately in 1.4% of patients, in particular metoclopramide and levosulpiride. Chronic use and abuse of these drugs lead to parkinsonism. They are harmful drugs with possible side effects at the heart level; in fact, they can lead to strong antagonism with D2 receptors [1], with the consequent onset of extrapyramidal syndrome. This, in turn, can lead to a prescriptive cascade with L-dopa in patients who are not suffering from Parkinson’s disease. It would be sufficient in these cases to stop the inappropriate drug to avoid the occurrence of tremors, bradykinesia, and stiffness. In addition, metoclopramide may have the properties and effects of class I antiarrhythmics [25].

Following the STOPP&START criteria, we stopped metoclopramide and levosulpiride, replacing them with more tolerated agents. Indeed, many over-the-counter drugs contain gentian, fennel, cumin, and green clay.

A total of two patients in our study were taking amiodarone, which is a potentially inappropriate drug, as it can cause changes in thyroid function and pulmonary fibrosis, following chronic administration [1,15].

A case of amiodarone-induced hepatotoxicity was described in an older patient after intravenous administration [26].

As for the proton pump inhibitors (PPIs), their use in recent years has increased; in our study, the prescriptions were 95 (46.3%); however, the rate of prescription was lower than those reported in the other literature studies [1,17]. We stopped pump inhibitors in 10 patients, as they were used in chronic therapy without existing common indications (prevention of bleeding in subjects treated with aspirin, chronic ulcerative disease).

Common side effects include headache, nausea, diarrhea, and rash [27]. PPIs may be associated with an increased risk of fractures, hypomagnesemia, Clostridium difficile infections, community-acquired pneumonia, and vitamin B12 deficiency [27].

In support of this, when ongoing therapy is not clear, the risk of side effects may outweigh the potential beneficial effects [27]. In addition, their use has been associated with an increased risk of death from acute renal failure and cardiovascular disease [27].

A relationship was found between exposure to PPIs and the risk of all causes of mortality. A thorough analysis of the death causes showed that taking PPIs was associated with an excess of mortality due to cardiovascular problems, chronic kidney disease, and upper gastrointestinal tract cancer. The problem was also observed in patients who did not have clinical indications for PPI intake, so it is preferable to have greater vigilance for the use of pump inhibitors [28].

Both typical and atypical antipsychotics were widely used in this study, although it is known that their excessive use should be avoided as a first line in the treatment of behavioral and psychological symptoms of dementia (BPSD). Their use is justified whenever other non-pharmacological options have not worked or in patients with auto- and hetero-aggression, with the consequent risk to themselves or others.

On the other hand, the use of antipsychotic drugs has raised concerns about the potential risk of sudden death, cerebrovascular events, and metabolic and hematological side effects. Careful monitoring of the QTc interval on the electrocardiogram (ECG) and serum electrolytes is also required.

However, antipsychotics are probably the best option for short-term treatments (6–12 weeks) for severe and persistent aggression [29,30,31,32,33,34].

In order not to use these drugs inappropriately, one should always assess whether the patient is taking the drug or not, whether the prescription was appropriate, and especially whether it is still necessary. A 25–50% reduction in the dosage of the drug every 1–2 weeks is advisable [32].

In our study, we used antipsychotics for 57.1% of outpatients and 79% of home patients. However, we reduced the dosage and tried to eliminate antipsychotics gradually.

We finally eliminated ten dangerous drugs for interaction, risk of triple whammy, serotonin syndrome, synergy, and pharmacodynamic antagonism. In particular, however, we plundered digital in two cases for the possible drug–disease negative interaction.

An 83-year-old patient took a digital dose > 0.125 mg but had a creatinine clearance of 28 mL/min/1.73 m^2^.

Another 98-year-old patient was taking digoxin 0.125 mg, which at the time of the visit presented nausea, loss of appetite, and anxiety. The dosage of serum digoxin showed values of 3 ng/mL, far above the normal range (0.9–2 ng/mL). The interruption of the treatment with digital allowed the complete recovery of the patient.

Eventually, it is necessary to check all the drugs taken by the patient at each visit, trying to deprescribe the drugs that are no longer necessary for their clinical management.

**Figure 6:**
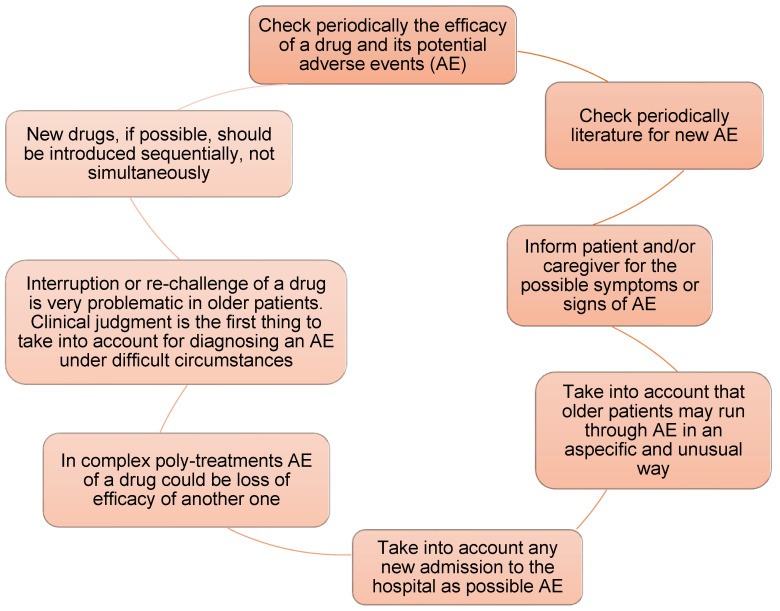
Tips for a safe prescribing in older patients.

Figure 6 offers practical suggestions to be taken into account when visiting a poly-treated patient [1].

The main challenge for the doctor remains mainly related to the lack of guidelines for managing comorbidities in poly-treated older patients [33].

The findings of our study are in line with other studies of the literature, where focus has been on the risk of polypharmacy in older patients, either in dementia or non-dementia patients. Furthermore, the evidence base for deprescribing in older people is gradually growing. Deprescribing tries to balance the potential benefit/harm ratio by systematically withdrawing inappropriate medications. There is also evidence that deprescribing procedures should start by withdrawing specific medications (i.e., antipsychotics, long half-life benzodiazepines) using individualized interventions [34,35]. 

Regarding other settings, in a previous paper on residential care patients, data obtained retrospectively from 972 residential care patients in a two-year time frame were in line with the present study, because the most frequent inappropriate drugs were anticholinergic drugs, tricyclics antidepressants, long half-life benzodiazepines, antipsychotics, and proton pump inhibitors [11]. 

Interestingly, general practitioners accepted the proposed changes to treatment.

## 5. Conclusions

In conclusion, we suggest how the process of deprescribing starts at the time of prescription, choosing rationally the therapy to be administered to patients. The present study suggests some important and final conclusions:

1. In our study, we showed an incredible number of over prescriptions; one should never prescribe a new drug without checking the other drugs that the patient already takes. It is always advisable to assess the risk/benefit ratio for each new drug.
2. We should periodically re-evaluate drug treatment, because older people often take drugs for several years without a real need; the collaboration among general practitioners, geriatricians, and pharmacologists is a fundamental step to this scope.
3. A rational approach to drug treatment consists in the personalization and simplification of therapy; we tried to do so in our study, stopping the administration of potentially dangerous drugs and changing them with more tolerated drugs. For example, the use of anticholinergics and benzodiazepines, especially long half-life benzodiazepines should be avoided the more the possible.
4. We should reduce the number of drugs administered as much as possible in order to improve compliance and maintain a high alert threshold to avoid potentially harmful interactions.
5. Priority should be given to the discontinuation of drugs with the lowest benefit/harm ratio and the lowest probability of adverse reactions due to withdrawal or rebound syndrome. For example, in our study, NSAIDs, alpha-blockers, and drugs with anticholinergic properties were discontinued.
6. Currently, there are no guidelines to properly address the comorbidities and management complexities of the patient. The Beers criteria (defining the use of potentially inappropriate drugs), and the STOPP&START criteria can be useful tools to this aim.
7. Every doctor should be aware of the possible cascade prescriptions, enemies of the older poly-treated patient.

In conclusion, we underline the need for such studies in wider populations, in order to appropriately guide prescriptions in older patients. This is going to be the new challenge on the future horizon.

## Figures and Tables

**Table 1:** Drugs with narrow/high therapeutic index [1].

Drugs with Narrow Therapeutic Index	Drugs with High Therapeutic Index
Aminoglycosides	Beta-blockers
Quinidine	Buspirone
Digoxin	Chlordiazepoxide
Phenytoin	Diazepam
Lidocaine	Ibuprofen
Procainamide	Lorazepam
Lithium salts	Oxazepam
Teophylline	Tetrahydrocannabidol
Vancomycin	
Dicumarols	

**Table 2:** Main characteristics of the sample.

	Outpatients	Home Patients	*p*	All the Sample
**N**	114	91		205
**Age, years**	80.2 ± 7.3	85.7 ± 6.4	**0.001**	82.7 ± 7.4
**Gender**				
**Men**	38 (33.4%)	31 (34.1%)		69 (33.6%)
**Women**	76 (66.6%)	60 (65.9%)		136 (66.4%)
**Education**	5.4 ± 2.9	4.8 ± 2.1	**0.09**	5.1 ± 2.4
**CIRS:**				
**Comorbidity index**	4.2 ± 1.5	4.8 ± 1.7	**0.008**	4.53 ± 1.17
**Severity index**	1.6 ± 0.2	1.7 ± 0.2	**0.001**	1.76 ± 0.85
**MMSE**	20.7 ± 3.5	16.4 ± 4.3	**0.001**	18.2 ± 3.9
**ADL**	2.9 ± 1.1	1.3 ± 1.05	**0.001**	2.23 ± 1.08
**IADL**	2.31 ± 1.2	0.74 ± 1.07	**0.001**	1.65 ± 1.13
**Main diagnosis**	x/114	y/91		x + y/205
Mixed dementia	15 (13.1%)	19 (20.8%)		34 (16.7%)
Alzheimer’s dementia	24 (21.1%)	17 (18.6%)		41 (20%)
Vascular dementia	19 (16.7%)	16 (17.5%)		35 (17.3%)
Frontotemporal dementia	3 (2.6%)	2 (2.2%)		5 (2.4%)
Lewy Body Dementia	1 (0.9%)	1 (1.1%)		2 (0.9%)
Parkinson-dementia	2 (1.7%)	1 (1.1%)		3 (1.5%)
MCI	9 (7.9%)	2 (2.2%)		11 (5.5%)
Parkinsonism	6 (5.3%)	4 (4.4%)		10 (4.4%)
Depression	11 (9.6%)	9 (9.9%)		20 (9.7%)
Previous stroke	7 (6.1%)	8 (8.8%)		15 (7.4%)
Other	17 (14.9%)	12 (13.2%)		29 (14.2%)
**Comorbidities**				
Heart disease (ischemia, hypertension, heart failure, atrial fibrillation)	99 (86.8%)	84 (92.3%)		183 (75.7%)
Osteoarthritis	87 (76.3%)	85 (93.4%)		172 (83.9%)
Diabetes	59 (51.7%)	78 (85.7%)		137 (66.8%)
COPD	65 (57.01%)	68 (74.7%)		58 (16.5%)
BPSD	59 (51.7%)	65 (71.4%)		123 (60%)
**Drugs**				
Cholinesterase inhibitors	62 (54.4%)	28 (30.7%)		90 (43.9%)
Memantine	32 (28.1%)	21 (23.1%)		53 (25.8%)
Cardiovascular drugs	99 (86.8%)	84 (92.3%)		183 (89.2%)
NSAIDs	18 (15.8%)	24 (26.4%)		42 (20.5%)
Antidiabetics	67 (58.7%)	70 (76.9%)		137 (66.8%)
Benzodiazepines	31 (27.2%)	38 (41.7%)		69 (33.6%)
Antipsychotics	65 (57.1%)	72 (79.1%)		153 (74.6%)
Other	8 (7.1%)	10 (10.9%)		18 (8.8%)
**Mean drugs used**	**9.8 ± 4.4**	**8.9 ± 3.5**	0.113	**9.4 ± 3.9**

ADL: Activities of Daily Living; IADL: Instrumental Activities of Daily Living; CIRS: Cumulative Illness Rating Scale; MCI: Mild Cognitive Impairment; MMSE: Mini Mental State Examination; COPD: Chronic Obstructive Pulmonary Disease; BPSD: Behavioral and Psychological Symptoms of Dementia; NSAIDs: Non-Steroidal Anti-inflammatory Drugs.

## Data Availability

Data available on request due to restrictions e.g., privacy or ethical.

## Supplementary Materials

The following supporting information can be downloaded at: https://www.mdpi.com/article/10.3390/geriatrics9020028/s1, Table S1: Potentially Inappropriate Medications (PIMs); Table S2: Inappropriate drugs for class and/or dosage; Table S3. Interactions = [number of drugs] × ([number of drugs] − 1)/2.
