# Supplementary material for: Deprescribing in Older Poly-Treated Patients Affected with Dementia
Source: Geriatrics (Basel). 2024 Feb 26;9(2):28. doi: 10.3390/geriatrics9020028 (PMC10961769; doi:10.3390/geriatrics9020028)
Supplement: Supplementary file 1 [file geriatrics-09-00028-s001.zip › geriatrics-2848624-supplementary.pdf]

Table S1. Potentially Inappropriate Medications (PIMs)

**Inotropic agents**	Digoxin - Daily dosage > 0,125 mg/day
**Long half-life benzodiazepines**	Diazepam, N-demethyl-diazepam, Chlordiazepoxide
**Tricyclic Antidepressants**	Amitriptyline, Imipramine
**Phenothiazines with piperidine structure**	Thioridazine
**Butyrophenones**	Haloperidol
**Atypical Antipsychotics**	Clozapine, Risperidone, Olanzapine, Quetiapine, Aripiprazol, Paliperidone
**Belladonna Alkaloids, semi-synthetic, quaternary ammonium compounds**	Butylscopolamine
**Antihistamines for systemic use**	Prometazine, Diphenhydramine
**Antihypertensive agents**	Methyldopa; Nifedipine;
**Antiarrhythmics**	Disopyramide
**α_1-adrenergic blockers**	Doxazosin, Terazosin
**Presynaptic α_2-agonists**	Clonidine
**Laxatives**	Sodium Picosulfate, Bisacodyl
**Antidiarrheals**	Loperamide
**Prokinetics**	Metoclopramide
**Class III antiarrhythmic drugs**	Amiodarone
**NSAIDs (Non-Steroidal anti-inflammatory drugs)**	Derivates of salicylic acid: Aspirin<br>Derivates of acetic acid: Indometacin<br>Derivates of propionic acid: Naproxene<br>Oxicam: Piroxicam, Tenoxicam

Table S2. Inappropriate drugs for class and/or dosage

**Benzodiazepines**	
	Alprazolam > 2 mg day
	Long half-life benzodiazepines (diazepam, chlordiazepoxide, clorazepate, N-demethyl-diazepam, clonidinium-chlordiazepoxide, flurazepam)
	Lorazepam > 3 mg day
	Meprobamate
	Oxazepam > 60 mg day
	Temazepam > 15 mg die
	Triazolam > 0.25 mg die
**Antihistamines**	
	Cyproheptadine
	Chlorpheniramine
	Diphenhydramine
	Hydroxyzine
**Stimulating laxatives**	Bisacodyl
**Antibiotics**	Nitrofurantoin

Table S3. Interactions = [number of drugs] x ([number of drugs]-1)/2 [1].

**Number of prescribed drugs**	**Interactions**
1	0
2	1
3	3
4	6
5	10
6	15
7	21
8	28
9	36
10	45
